# An Atypical Presentation of Central Sleep Apnea in the Context of Bariatric Surgery: A Case Report

**DOI:** 10.7759/cureus.90228

**Published:** 2025-08-16

**Authors:** Serena Sanchez, Jennifer Brock

**Affiliations:** 1 Internal Medicine, White County Medical Center, Searcy, USA; 2 Sleep Medicine, Searcy Medical Center, Searcy, USA

**Keywords:** bariatric surgery, bilevel positive airway pressure (bipap), central sleep apnea (csa), complex sleep apnea syndrome (compsas), continuous positive airway pressure (cpap), obstructive sleep apnea (osa), restless leg syndrome (rls)

## Abstract

Obstructive sleep apnea (OSA) is considerably associated with obesity due to increased upper airway tissue deposition, increased upper airway resistance, and reduced airflow. Bariatric surgery is a consideration in those with severe obesity to facilitate weight loss and aid OSA symptoms. However, postoperatively, patients are at an increased risk of developing central apneic events, thereby leading to complex sleep apnea syndrome (CompSAS). The management of CompSAS is nuanced and can involve the utilization of continuous airway pressure (CPAP), bilevel positive airway pressure (BiPAP), and/or adaptive sero-ventilation (ASV). Overall, the approach to treating CompSAS remains multifaceted, and numerous considerations should be made in terms of managing post-bariatric surgery patients and their sleep-disordered breathing conditions.

## Introduction

Obstructive sleep apnea (OSA) is highly correlated with an increased body mass index (BMI) due to augmented neck circumference, enlarged pharyngeal soft tissues, and impaired upper airway patency [1]. As a result of decreased ventilation, repeated arousals and episodic oxygen desaturation occur during sleep, which leads to significant daytime hypersomnolence, an increased risk of cardiovascular disease, and cognitive dysfunction [1]. Current OSA treatment options aim to reduce upper airway resistance and prevent obstruction, which can include oral appliances, continuous positive airway pressure (CPAP), and upper airway surgery [2]. However, in the severely obese, the current treatment methods do not address the obesity itself nor the anatomical variations associated with obesity. Observational studies have suggested that weight loss resulting from bariatric surgery leads to substantial remission of OSA symptoms in 60-80% of patients [3]. However, other studies have found that the use of bariatric surgery, when compared to conventional weight loss, does not statistically result in a greater reduction of the apnea-hypopnea index (AHI) despite significant weight loss [2]. Therefore, OSA is likely complex in its pathogenesis, which requires a more nuanced approach to its treatment rather than viewing the condition purely mechanistically. 

Central sleep apnea (CSA) can be defined as a transient cessation of respiration initiated by the pontomedullary region of the brain, resulting in a brief absence of ventilatory output during sleep [4]. CSA can result in both hypoventilation and hyperventilation and is associated with neuromuscular conditions (e.g., amyotrophic lateral sclerosis), spinal cord injuries, and CNS depressants (e.g., opioids) [4]. Although CSA is less prevalent compared to OSA, the two conditions can coexist, and patients may exhibit features from both conditions [4]. 

Complex sleep apnea syndrome (CompSAS) is defined as the development or persistence of central apneic events or hypopneas that occur with the initial application of CPAP and has been associated with developing after undergoing various upper airway surgical interventions [5]. Previously, this phenomenon was referred to as “treatment-emergent central apnea” [6]. Specifically, patients with CompSAS exhibit features similar to OSA, but also display breathing patterns identical to those of CSA [6]. The mechanisms underlying CompSAS are still not fully understood, including the extent of the cause-and-effect relationship associated with CompSAS [5-6]. Numerous mechanisms are thought to contribute to chemoreceptor hypersensitivity and ventilatory instability [7] leading to CompSAS, including CPAP over-titration. Specifically, the over-titration of CPAP may lead to a drop in cardiac output, which promotes ventilatory instability by lengthening circle time, thereby leading to a worsening sleep quality of CPAP users [5]. These changes from sleep to wake and vice versa lead to further ventilatory instability, thereby causing oscillations in arterial carbon dioxide partial pressure (PaCO₂), which may fall below the apneic threshold and result in central apneas [5]. Incidentally, although CPAP can act as a trigger for central apneic events, it remains the first-line treatment for CompSAS, with continued use and, when necessary, consideration of bilevel positive airway pressure (BiPAP) and/or adaptive servo-ventilation (ASV) [6].

## Case presentation

The patient was a 58-year-old woman with a medical history of hemochromatosis and moderate obstructive sleep apnea (OSA; AHI 17, diagnosed 3/2015, BiPAP compliant) who presented to the sleep medicine clinic in January 2024 with worsening sleep quality following a recent sleeve gastrectomy. Before the bariatric surgery, the patient weighed 275 pounds, and after the surgery, the patient weighed 183 pounds. Upon presentation and shortly after undergoing the bariatric surgery, the patient experienced xerostomia, daytime hypersomnia, reports of increased mask leak, and an increase in restless leg syndrome (RLS) symptoms, with an International Restless Legs Syndrome Study Group (IRLS) rating scale of 20/40, indicating moderate symptoms. The patient had reiterated that she had been BiPAP compliant with no adjustment of settings since the 2015 initiation, given adequate daytime functioning. After reviewing the BiPAP device, it was revealed that the patient likely started to experience CompSAS, accompanied by worsened nocturnal sleep and daytime sleepiness.

Given the suspicion for CompSAS, her BiPAP was transitioned to fixed CPAP at a pressure of 8 cm H₂O. Her AHI improved markedly, decreasing from a moderate 17 in 2015 to 1.8 in 2024. Overnight pulse oximetry demonstrated a normal oxygen saturation of 94%, despite the transition from a full-face to a nasal mask for patient comfort. IRLS improved to 7/40, indicating mild and controlled symptoms, presumed secondary to appropriate sleep apnea management. The patient's ferritin level was confirmed to be within the appropriate range at 372 ng/mL, indicating reasonable control of hemochromatosis and RLS. Since transitioning to CPAP at a lower pressure, the patient has reported an Epworth Sleepiness Scale (ESS) score of 5/24 (within the normal range), along with improved sleep quality, reduced daytime hypersomnia, and better subjective control of limb movements. 

## Discussion

In this case, the patient’s preoperative BiPAP had effectively controlled her OSA since its initiation in 2015. However, following sleeve gastrectomy with a reported weight loss of approximately 90 pounds, she developed worsening hypersomnolence along with new central apneic events consistent with CompSAS. Given the patient’s weight loss, it can be assumed that changes in upper airway anatomy contributed to decreased soft tissue, reduced predisposition to pharyngeal collapse, improved pharyngeal patency, and increased airflow [1]. As a result, the pre-surgical fixed bilevel pressures may have become excessive, thereby reducing PaCO_2_ below the apneic threshold, likely triggering central apneas [5]. Once CompSAS was suspected, the patient’s setting was reduced to a fixed CPAP pressure of 8 cm H₂O, which was likely more appropriate given the physiological changes associated with her postoperative weight loss. This change to static pressure resulted in a considerable reduction in her AHI and a significant improvement in her sleep quality. Furthermore, following the pressure adjustment, the patient’s ESS and IRLS scores improved, supporting the likelihood that apneic events were contributing to her fatigue and RLS symptoms. 

There are other considerations concerning this patient’s presentation that should be considered as differential contributing factors. First, despite the patient’s weight loss, residual craniofacial or airway abnormalities may continue to contribute to OSA. Furthermore, despite improvements in AHI after bariatric surgery, most patients will likely continue to require treatment (e.g., PAP) for OSA [8]. Second, medications such as benzodiazepines, opioids, and certain antidepressants act on the central nervous system and are thought to contribute to sleep-disordered breathing and promote CSA [9]. An extensive review of the patient’s home medications was performed, with no obvious contributing CSA medications discovered. It was not specified whether the patient was taking pain medications, such as opioids, following the recent surgical intervention. Lastly, the patient’s RLS was initially classified as moderate and may have contributed to sleep fragmentation. However, the IRLS score improved significantly after the adjustment of PAP pressure settings, suggesting that the RLS was exacerbated by apneic events rather than serving as the primary cause. 

## Conclusions

This case presentation highlights the interplay between obesity, weight loss, and sleep-disordered breathing, particularly in the development of CompSAS after bariatric surgery. While OSA is associated with obesity and typically improves with weight loss, clinicians should be aware of potential outcomes, such as the development of CSA, after resuming PAP therapy post-surgery. Though CSA post-bariatric surgery is uncommon, the need to consider post-bariatric repeat sleep assessments and/or polysomnography (PSG), especially when central events are suspected. Furthermore, re-evaluation of unchanged PAP settings should be considered during post-surgical follow-up to avoid over-titration, particularly after weight loss. Thus, the importance of individualized therapy following significant physical and physiological changes, such as in bariatric surgery, is crucial in managing pre-existing OSA and potentially avoiding central events. The successful down-titration of pressure resolved the patient’s hypersomnolence and underscores the importance of appropriate titration in mitigating symptoms and restoring sleep quality. 
